# A Combined Fully Convolutional Networks and Deformable Model for Automatic Left Ventricle Segmentation Based on 3D Echocardiography

**DOI:** 10.1155/2018/5682365

**Published:** 2018-09-10

**Authors:** Suyu Dong, Gongning Luo, Kuanquan Wang, Shaodong Cao, Qince Li, Henggui Zhang

**Affiliations:** ^1^School of Computer Science and Technology, Harbin Institute of Technology, Harbin 150001, China; ^2^Department of Radiology, The Fourth Hospital of Harbin Medical University, Harbin 150001, China; ^3^School of Physics and Astronomy, University of Manchester, Manchester, UK; ^4^Space Institute of Southern China, Shenzhen, Guangdong, China

## Abstract

Segmentation of the left ventricle (LV) from three-dimensional echocardiography (3DE) plays a key role in the clinical diagnosis of the LV function. In this work, we proposed a new automatic method for the segmentation of LV, based on the fully convolutional networks (FCN) and deformable model. This method implemented a coarse-to-fine framework. Firstly, a new deep fusion network based on feature fusion and transfer learning, combining the residual modules, was proposed to achieve coarse segmentation of LV on 3DE. Secondly, we proposed a method of geometrical model initialization for a deformable model based on the results of coarse segmentation. Thirdly, the deformable model was implemented to further optimize the segmentation results with a regularization item to avoid the leakage between left atria and left ventricle to achieve the goal of fine segmentation of LV. Numerical experiments have demonstrated that the proposed method outperforms the state-of-the-art methods on the challenging CETUS benchmark in the segmentation accuracy and has a potential for practical applications.

## 1. Introduction

The assessment of left ventricle (LV) function on the echocardiography plays a key role in the diagnosis of heart disease. The LV segmentation based on echocardiographic images is an essential step for LV function assessment in terms of characterizing the ventricular volume, ejection fraction, wall motion abnormalities, and myocardial contractility [1, 2]. Compared to the traditional 2D echocardiography, 3D echocardiography (3DE) allows a real-time 3D visualization of the heart [3]. However, due to some intrinsic limits such as low signal-noise ratio, low spatial and temporal resolutions, and presence of motion artifacts, the fully automatic segmentation of the LV in 3DE is still an open and challenging task [4]. Moreover, manual segmentation is time-consuming and prone to subjective variability [5]. Therefore, an automatic and accurate LV segmentation method is desirable for accessing the LV function. Current methods for LV segmentation on echocardiography can be classified as deformable models, statistical models, and machine learning methods [6].

Deformable models are widely used for the LV segmentation in echocardiography [7–13]. With this method, an energy function is defined and minimized for accurate detection of the LV boundary. The widely used deformable models can be divided into snake model and level-set model, which are based on the boundary and region information, respectively [14, 15]. Usually, some new constraints are integrated into the common energy function to achieve a more accurate segmentation [10, 12, 16]. Although they are useful in LV segmentation on multimodality medical image data [10, 17, 18], these models have intrinsic limitations as they depend much on the initialization and image quality. In general, good initialization is necessary, especially for ultrasound images, which are vague and noisy. Currently the common initialization method of LV segmentation is manual or semiautomatic. Therefore, an accurate and automatic initialization method is the key for fully automatic LV segmentation.

Statistical models, such as active shape model (ASM) [19, 20] and active appearance model (AAM) [21], are based on the statistical information from large labeled data from experts [22]. The statistical information from the labeled data is modeled using some parameters, which are mainly based on contour borders and image textures information in image. Recently, the AAM and ASM have been widely applied to the LV segmentation problem of echocardiography [23–28]. However, due to the dependence on the large number of annotated images, initialization, and assumption of shape and appearance [29–32], statistical models exhibit clear limitations on the automatic segmentation of LV.

Different from statistical models, machine learning methods do not depend on the assumption of shape and appearance. Machine learning methods have demonstrated excellent performance in natural image segmentation. Compared to the traditional machine learning methods, which are based on the hand-crafted features, deep convolutional networks achieved milestone segmentation results in natural images [33–36]. Inspired by the remarkable success in natural image segmentation, recently some studies have successfully implemented the deep convolutional networks for LV segmentation [14, 17, 29, 37, 38]. However, compared to the natural image segmentation, the LV segmentation of 3DE is limited by a lack of large training datasets and low signal-to-noise ratio. So far, few researchers try to formulate the LV segmentation task on 3DE into a deep learning task. Some research combined deep learning method and deformable model to segment LV on cardiac MR images [17, 38], in which deep learning methods were employed to produce a rectangle to detect the region of interest (ROI) of LV, and then other postprocessing method was used to make a final segmentation of LV.

Different from these researches, we proposed a new fully automatic method which employed fully convolutional networks and deformable model for LV segmentation on the 3DE. In this work, we advance our preliminary attempt on LV segmentation of 3DE [39]. The main contributions are following points:

(1) We formulated the fully automatic LV segmentation task into a coarse-to-fine framework, which includes the coarse segmentation based on deep learning technology and fine segmentation based on the 3D snake respectively.

(2) We employed data augmentation method and the transfer ability of deep convolutional networks between the natural image and 3DE and proposed a new fusion network structure to further improve the coarse segmentation results.

(3) Based on the coarse segmentation results, we proposed a new initialization method utilizing the relation between the spatial position and region sizes of LV in the coarse results and further improved the traditional 3D snake model based on the spatial regularization to generate the fine segmentation results. Besides, compared to the purely end-to-end deep learning method, the proposed method has high interpretability.

(4) Numerical experiments have been carried out to demonstrate that the proposed method outperforms the state-of-the-art methods on the challenging CETUS benchmark in the segmentation accuracy and has potential to be applied into clinical application.

The manuscript is structured as follows. In Section 2, our method is described in detail. In Section 3, some experimental results are presented, including a comparison with state-of-the-art methods. In Section 4, we discuss the results and characteristics of the proposed framework. Finally, we conclude this work and discuss its future applications in Section 5.

## 2. Method

**Figure 1:**
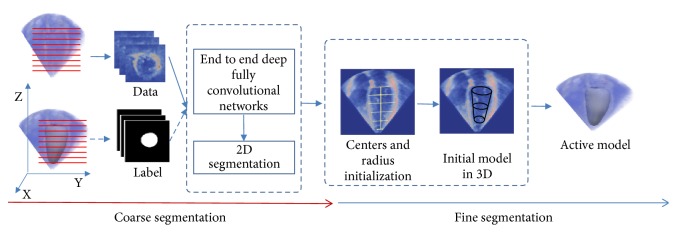
The overview of the proposed framework from coarse segmentation to fine segmentation.

As shown in Figure 1, we formulate the LV segmentation problem into a coarse-to-fine framework. First, to get enough training data for deep learning, we employed an appropriate data augmentation method based on the conversion from 3D volume to 2D slices. Second, an improved deep fully convolutional network (FCN) based on feature fusion and transfer learning was applied to make initial segmentation of all original 2D slices from 3DE. And then, based on the initial segmentation results, the initialization models for the deformable model were constructed. Finally, an improved 3D snake model was used to segment 3DE. The details of the proposed method are as follows.

### 2.1. Coarse Segmentation

We used the deep FCN in [33], which does not contain fully connected layer and uses skip structure for the first time, to achieve the initial segmentation. FCN can provide an accurate localization and initial shape of LV for the further fine segmentation. However, due to limited training datasets, few researchers try to adopt the deep learning technology to address the LV segmentation problem on 3DE. To overcome the problem of insufficient training datasets, we adopted the transfer learning method to initialize the deep FCN model. Additionally, to improve the segmentation accuracy of deep FCN, we proposed a new FCN structure based on the feature fusion across different layers and residual module. Subsequently, we introduce the coarse segmentation method in the aspect of data augmentation and transfer learning and improved the structure of the FCN, respectively.

#### 2.1.1. Date Augmentation

The CETUS benchmark [3] is believed to have the largest open accessible 3DE datasets. Hence, we adopted the CETUS benchmark datasets to train and validate the FCN model. The CETUS dataset includes 45 3DE subjects, which were acquired from three ultrasound machines of three different vendors. The subjects were divided into 15 training subjects and 30 test subjects, and each subject included two labeled volumes in the end-systole (ES) and end-diastole (ED) frames.

However, due to a small training dataset, the data augmentation is necessary for training deep learning model. To solve this problem, we employed an appropriate data augmentation method based on the conversion from 3D volume to 2D slices. As shown in Figure 1, the 3D volumes were sliced along the Z axis into 2D images, and the 3D mesh of ground truth was simultaneously sliced to match the corresponding 2D images. In this way, all the 2D images were used as the input, so that the number of input samples is increased by N times of the number of 3DE samples (N is the height of Z axis). In this way, we not only get more training samples, but also save the number of parameters on deep learning model (because we convert the input format from 3D to 2D).

Based on the generated 2D images datasets, some traditional data augmentation methods (such as rotation and resizing) can be adopted to achieve further data augmentation to get lots of training images for the following deep FCN training. Note that we copy the channel of an original 2D image three times to generate a 2D image with three channels to fit the input of the FCN for the following transfer learning technology. Because the weights, which are used for transfer learning, are from VOC dataset. The images of this VOC dataset are RGB images, and every image has three channels. Hence, the only way is to copy the channel of an original 2D image three times to make sure the same number of channels to make the transfer learning work well. If we modify the FCN to deal with a single channel, we are unable to transfer the weights properly from the pretrained FCN model on VOC datasets.

#### 2.1.2. Transfer Learning Based on VOC Datasets

**Figure 2:**
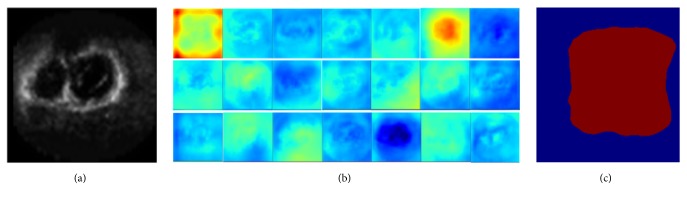
The feature maps of the last layer of FCN model with pretrained weights on VOC datasets, adopting the 2D echocardiography as input. (a) The original input 2D slice. (b) 21 feature mappings on the last layer. (c) The final output result of FCN.

Though the number of training samples was increased by the proposed augmentation method, training the deep FCN model from scratch is difficult. Inspired by some related researches, which adopt the transfer learning technology to overcome the limitation of datasets and avoid the overfitting problems [42–44], we transfer the weights from pretrained FCN model [33] on VOC datasets (which were collected from photo-sharing web site and include 1464 RGB images for pixel-level segmentation) [45] to initialize FCN model and then fine-tune it on augmented echocardiography datasets. To prove the transferability of the pretrained FCN model between the RGB and echocardiography datasets, we used the pretrained FCN model on VOC datasets to predict the pixel classes of the 2D images from 3DE directly.  Figure 2 shows the 21 feature maps (output of the last layer in FCN) and the final output result of FCN model with pretrained weights on VOC datasets. We can see that the regions of LV in 2D images have higher response values and can be extracted as a single class. This result proves that the pretrained model has transferability.

#### 2.1.3. Fully Convolutional Networks Using Features Fusion

**Figure 3:**
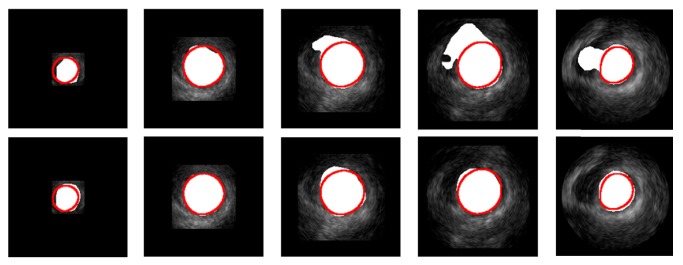
Comparing ground truth (red circles) with the segmentation results of original FCN (first row) and improved FCN (second row) of Patient 15 (which was used as validation subject) from the CETUS benchmark [3]. The slices from left to right correspond to the LV parts from apex to base.

We adopted the FCN in [33] as our basic networks structure and modified the number of channels according to the number of classes (from 21 to 2) in all deconvolution layers. The 21 classes are aeroplane, bird, bicycle, boat, bottle, bus, car, cat, chair, cow, dining table, dog, horse, motorbike, person, potted plant, sheep, sofa, train, TV/monitor, and background [45]. The 2 classes are LV and background. We used the pretrained models on VOC datasets to initialize all the convolutional layers of the FCN and fine-tuned the whole networks on the adopted echocardiography datasets. The details about networks training are shown in Section 2.1.5. The first row of Figure 3 shows the LV segmentation results using the FCN model trained through transfer learning (using pretrained models), which shows the potential of FCN based on transfer learning. However, to achieve more accurate segmentation, we proposed the new structure to improve the coarse segmentation accuracy.

**Figure 4:**
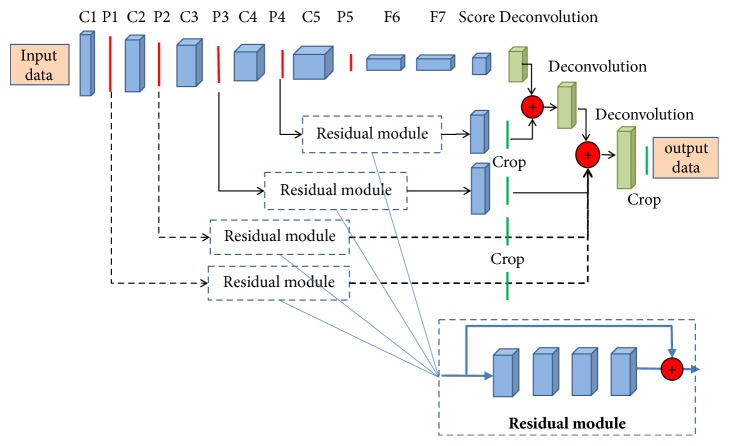
The structure of the improved FCN. Based on original FCN in [33], we added two streams from the first two pooling layers (pooling 1 and pooling 2) to the final deconvolution layers, and the residual modules are added to every skip stream to improve the ability of feature representation.

Inspired by the feature fusion strategy (it is also called skip structure) [33, 46–48] and residual networks [35], we proposed a new feature fusion method with residual connections. The new proposed method is based on an assumption that the feature fusion strategy can combine the feature maps in different levels (the low level in the bottom layers and the high level in the top layers), which are complementary to each other to boost the segmentation accuracy. Additionally, we employed the residual module with identity mapping [49] to encode the mid-layers features, based on the characteristic that the residual networks enable effective backward propagation of the gradient through the identity mapping and achieve fast convergence as well as good feature representation [50]. This characteristic is very important for training relatively complex convolutional neural networks (CNN). As shown in Figure 4, the parts with dotted lines denote the added streams and modules on the original FCN in [33]. In brief, we added two streams from the first two pooling layers (pooling 1 and pooling 2) to the final deconvolution layers, and the residual modules are added to every skip stream to improve the ability of feature representation. The adopted residual module includes 4 convolutional layers, and each layer includes 2 kernels with 3*∗*3 kernel size following by the BN layer [51] and Relu activation layer (rectified linear units f(x)=max(0, x)). Additionally, because only the LV regions and background were classified in our study, hence, in all deconvolution layers we employed two deconvolution kernels to generate the final two probability feature maps instead of 21 feature maps in the original FCN.

#### 2.1.4. Loss Function

For 2D echocardiography images, the size of LV foreground is usually much smaller than the size of background. Hence, the number of pixels in LV region and the number of pixels in background are heavily imbalanced. Here, given an image *I* and its ground truth* y*, a weighted cross-entropy loss function is used to balance LV region and background classification as follows:

(1) Loss=−a∑i=1Y+log⁡Pyi=1 ∣ I;w−1−a·∑i=1Y−log⁡Pyi=0 ∣ I;w

where *Y*_+_ and *Y*_−_ denote the numbers of pixels belonging to the foreground (LV region) and background, respectively, in the ground truth, a= *Y*_−_/(*Y*_+_ + *Y*_−_), *P* denotes the probability of predicted classification, and *w* means the weights of trained networks.

#### 2.1.5. Network Training

To utilize the pretrained FCN model and the advantage of added residual modules well, we adopted the two-stage manner to train the proposed networks. Firstly, we transferred the pretrained FCN model to initialize the parameters in the whole networks except for the deconvolution layers and the added residual modules. And then, we fixed the parameters of the layers with pretrained initialization and trained the deconvolution layers and the added residual modules; in this way, the deconvolution layers and the added residual modules can be relatively better pretrained for initialization. Finally, we fine-tuned the whole networks to get the model for LV coarse segmentation. The second row of Figure 3 shows the segmentation results of 2D slices from the apex to the base of LV using improved FCN based on features fusion and weighted cross-entropy loss function. Compared to the traditional FCN, the improved FCN can generate more accurate segmentation results. In some cases, the proposed method can discard the parts which are not belonging to the LV, and this is critical for the following fine segmentation step. And more experiments results will be shown in Section 3 to prove the superiority of the proposed network.

### 2.2. Fine Segmentation

Figure 3 displays the segmentation results in the coarse segmentation stage. Based on the coarse segmentation results, we proposed a fine segmentation method based on 3D initialization and the 3D snake model [52]. Then, we introduce the fine segmentation method in the aspects of the 3D initialization and improved 3D snake model.

#### 2.2.1. Initialization

**Figure 5:**
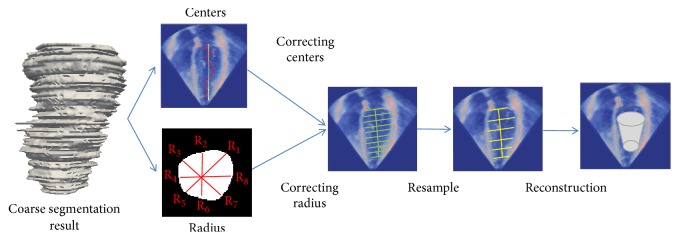
The pipeline of automatic 3D model initialization for active deformable model. Based on the segmentation result of improved FCN, we corrected the center and the scale of LV region and then reconstructed the 3D mesh based on the resampling method.

A good initialization is critical for LV segmentation on 3DE, which is low signal-noise ratio. Some similar works in [17, 38] have proved the feasibility of the 2D initialization based on deep learning for the segmentation task on MR images. Here we proposed an automatic initialization method on 3D space for LV segmentation on 3DE. In general, the initialization task consists of two subtasks: the center localization and the scale estimation of LV region. As shown in Figure 5, the improved FCN has advantages. It is able to get the relatively good segmentation results on 2D slices. To achieve the good center localization in 3D space, firstly, LV centers were estimated through averaging the coordinate values of all foreground pixels on coarse segmentation results in every slice directly. However, as we can see in Figure 5 (the red curve), the computed centers were noncollinear in 3D space. This problem leads to the misalignment between the adjacent slices and reduce the quality of constructed 3D initialization model. Hence, to achieve more accurate center point localization, as shown in Figure 5, the widely used quadrature curve fitting method [17] was used to correct the estimated centers to avoid the misalignment.

The scale estimation of LV region is on the basis of the assumption that the shape of LV in a 2D slice approximates a circle. This assumption is beneficial to LV initialization, which has been proved in [4]. Hence the radiuses of the LV region were computed using the formula *R* = (*R*_1_ + …+*R*_8_)/8, where the *R* denotes the average of radiuses in equal angles' samples as shown in Figure 5.

After the center localization and the scale estimation of LV region, we finally obtained some LV contours on slices along the Z axis. To achieve highly efficient model representation, the 3D mesh was reconstructed based on the resampling method. We equally resampled the contours according to the formula *S*_*i*_ = *B*_1+*i∗k*_, where *S* denotes the resampled contours set, *S*_*i*_ denotes the *i*th sampled contours along the *Z* axis, and *k* denotes the sampling interval,* B* denotes original contours set along the *Z* axis. Finally we reconstructed the initial 3D mesh model through the method in [53].

#### 2.2.2. Active Deformable Model

Based on the initial 3D mesh, an improved 3D snake model was proposed to segment LV from 3DE. Analogous to two-dimensional snake [52], our improved 3D snake was also based on the minimization of energy function and was optimized by gradient descent. Although similar in some ways, an improved energy function is designed in our method. In the proposed 3D snake model, the initialization 3D model obtained from the previous step was used as a 3D spatial regularization constraint for the proposed energy function. Compared to the regularization technology in [17, 38], the proposed 3D spatial regularization technology can appropriately limit the free degree of deformation, to avoid boundary leak between the LV and left atrium (LA) which is a common difficulty in the 3D LV segmentation tasks [14], because the LV and LA are adjacent and have similar pixels intensity. Additionally, the boundary leak due to the presence of papillary muscles can also be avoided.

The final 3D mesh of LV was got when the minimum of energy function was reached. The three-dimensional contour and energy function are defined as the following formulas, respectively:

(2) Xu=xu,yu,zu,

(3) E=∫12α∂X∂u2+β∂2X∂u22+δEextX+ηEinitXdu

where the first term and second term in (3) are internal energy and external energy, respectively. Same as the traditional snake model,* X*′ and* X*^*”*^ are the first-order term and second-order term of *X*, respectively, and they are controlled by parameter *α* and *β*. The first-order term makes the mesh smooth and the second term makes the mesh continuous. The third term *E*_*init*_(*X*) = |*X* − *X*_*init*_|^2^ was the spatial regularization term, in which *X* is the current mesh and *X*_*init*_ is the initial mesh. In this way, we not only can get a relatively finer mesh but also can avoid the absurd deformation due to boundary leak.

## 3. Evaluation and Results

### 3.1. Dataset

The proposed method has been tested by using the challenging CETUS benchmark datasets [3]. The ground truth is accessibly open for training set, but not for test set. Hence the final evaluation results of test set are from the official online evaluation system.

However, to train and evaluate deep FCN model for coarse segmentation, we divided the original 15 training subjects into 12 training subjects (24 volumes) and 3 validation subjects (6 volumes). As described in Section 2.1, we converted the 30 volumes into 5362 2D images; furthermore we used the traditional data augmentation method to rotate and resize the initial 2D images for 10 times randomly to get final 53620 images, in which approaching 45160 and 8460 images were used for training and evaluating deep FCN, respectively.

### 3.2. Setting

In the coarse segmentation stage, the proposed FCN adopted the training setting similar to that in [33]. We set learning rate 10^−4^ in the type of linear decreasing after every 1000 iterations, the momentum 0.99, the batch size 256, and the max iteration number 10000, respectively. The networks did not use any regularization such as L1, L2 regularization and dropout. Additionally, the initialization of network weights is crucial because of that it directly affects the convergence speed and effectiveness. In order to solve this problem, the pretrained weights coming from FCNs (was trained by in VOC datasets) were used to initialize our network. As described in Section 2.1, we first pretrained the deconvolution layers and residual modules fixing the other layers for first 1000 iterations and then trained the whole networks during the other 9000 iterations. In the fine segmentation stage, to guarantee the repeatability of the shown experiment results, we set the parameters of improved 3D snake *k* = 10, *α* = 0.1, *β* = 0.2, *δ* = 0.1, and *η* = 0.1, respectively, though parameters are robust in the most of cases.

In our study, the proposed FCN was implemented based on the widely used Caffe framework [54]. The whole experiment was performed on NVIDIA Titan X GPU.

### 3.3. Metrics

We adopted official metrics to evaluate the proposed method [3], such as the mean surface distance (d_m_), Hausdorff surface distance (d_h_), and modified dice similarity index (D*∗*), using which we compared the accuracy of our method with the ground truth from official CETUS benchmark [3].* S* and *S*_*t*_ denote the surface from the proposed method and surface from ground truth, respectively. Mean surface distance measures the mean distance between *S* and *S*_*t*_ and it can be computed by

(4) dm=d−S,St+d−St,S

(5) dh=max⁡min⁡S,St,min⁡St,S

Here, $\overset{-}{d}\left(S,S_{t}\right)$ denotes the mean distance between every voxel from *S* and the closest voxel from *S*_*t*_; $\overset{-}{d}\left(S_{t},S\right)$ is computed in analogous way. As shown in (5), the Hausdorff surface distance measures the local maximum distance between *S* and *S*_*t*_. Modified dice similarity index measures the overlap of surface and is computed by *D∗* = 1 − 2(*V*∩*V*_*t*_)/(*V* + *V*_*t*_).* V* and *V*_*t*_ denote the volume from proposed method and ground truth, respectively.

Besides, the performance of our method was also measured by official metrics, the modified correlation (corr*∗*), and standard deviation (std) in terms of end-diastolic volumes (EDV), end-systolic volumes (ESV), and ejection fractions (EF). EF is calculated by

(6) $$\begin{array}{c} EF=\frac{\left(EDV-ESV\right)}{EDV} \end{array}$$

Additionally, to evaluate the accuracy of coarse segmentation, we adopted the traditional metrics for 2D images segmentation [33], the pixel accuracy (acc), mean accuracy (mean acc), and mean IOU. The IOU measures the region intersection over union for every class, and the mean IOU is the mean value of different classes of IOU [33]. The three metrics are calculated by

(7) acc=∑inii∑iti

(8) meanacc=∑inii/ti2

(9) meanIOU=1/2∑iniiti+∑jnji−nii

where *n*_*ij*_ is the number of the pixels of class *i* predicted to class* j*; *t*_*i*_ denotes the pixels number of class* i*.

### 3.4. Results

To prove the potential and superiority of the proposed coarse-to-fine framework, we present and discuss the experiment results in two aspects: the segmentation accuracy and clinical validation.

#### 3.4.1. Segmentation Accuracy

**Figure 6:**
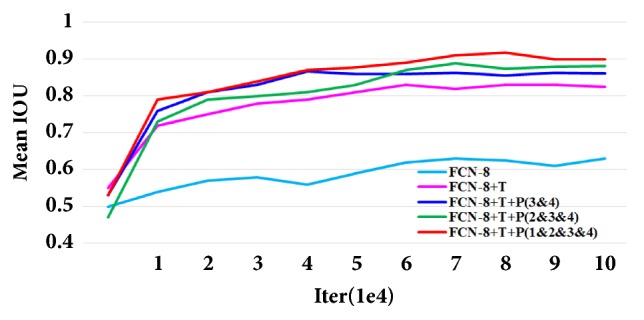
Different types of FCN's mean IOU curve along with increasing iterations in validation set.

(*1) Performance of FCN in Coarse Segmentation. *In order to better understand the importance of the improved FCN in our study, we compared the segmentation accuracy in terms of pixel accuracy, mean accuracy, and mean IOU. By using improved FCN, we obtained the initial segmentation results (coarse segmentation). To achieve clear expression and comparison, we define abbreviation T as the transfer learning based pretrained weights on VOC datasets; P_N_ denotes the added skip stream with residual modules from Nst pooling layers to the deconvolution layers. As shown in Table 1 and Figure 6, compared to FCN trained without pretrained model, the FCN trained with pretrained model from VOC datasets through transfer learning technology achieves significant improvement. These results prove the importance of the proposed transfer learning strategy. Additionally, as shown in Figure 6, with the increase of the training number, network using transfer learning and residual modules achieves higher accuracy and more rapid convergence than the FCN trained from scratch. Besides, we also can find that the proposed method achieves higher accuracy and faster convergence when it has more skip streams. The best results (pixel accuracy: 0.991; mean accuracy: 0.981; mean IOU: 0.918) were achieved by FCN+T+P (1&2&3&4) using the transfer learning and fused four skip streams. This observation shows that the fusion of low-level features in the bottom layers and high-level features in the top layers can achieve satisfactory performance in the 3DE semantic segmentation application.

Besides, to evaluate the advantage of the proposed FCN, we conducted the same evaluation using the state-of-the-art medical image segmentation model (U-net [43]). For transfer learning on the U-net, we used the pretrained model provided by [43] to initialize the U-net and then fine-tuned it on the adopted training dataset for 9000 iterations. Besides, to fit the data input format of U-net, we adopted the original one-channel images as the input. As shown in Table 1, the adopted FCN model outperforms the U-net on the two cases (the model trained from scratch and the model trained based transfer learning). These results prove the superiority of the adopted FCN model. Additionally, the Refine-net [45] achieves the best performance on the natural segmentation task. However, the Refine-net is very deep and complex, which cannot fit the current data scale on 3DE. Hence, we do not try to evaluate the performance of Refine-net on LV segmentation task on 3DE.

(*2) Performance of Fine Segmentation.* In this section, the final fine segmentation performance is evaluated through the official online evaluation system [3], and we compared the segmentation results with state-of-the-art methods (the results of the five state-of-the-art methods come from the official reports in [3]) in terms of d_m_, d_h_, and D*∗*. To show the superiority of the proposed initialization method based on the coarse segmentation using FCN, we conducted the experiments with varying initializations while keeping the traditional deformable model. As shown in Table 2, compared with the traditional initialization method [27], the proposed 3D model initialization method based on the FCN improves the final LV segmentation results. Besides, we also evaluated the proposed method. The evaluation and comparison results are shown in Table 3. We can see that the proposed method distinctly achieved the best segmentation results in mean surface distance and modified dice metric.

**Figure 7:**
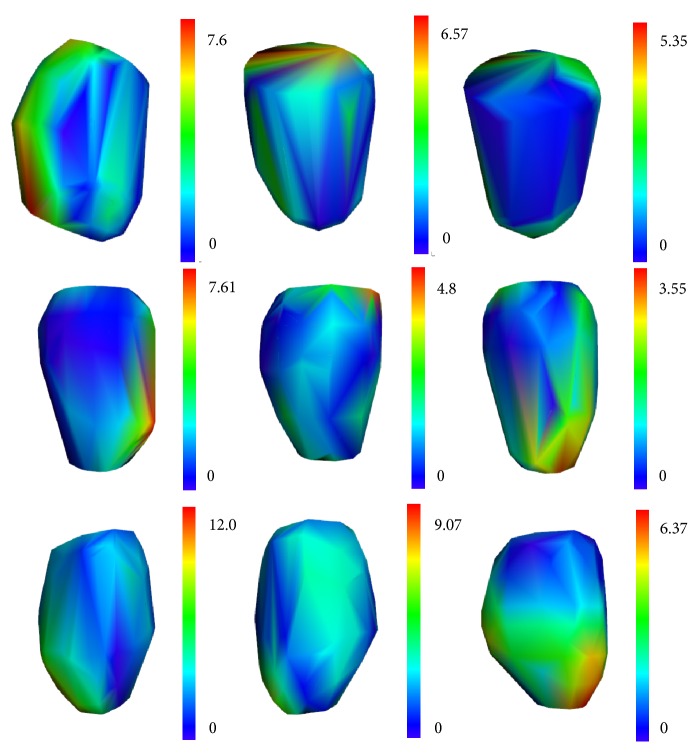
The segmentation results of three different subjects (which all are test subjects) of improved FCN (the first column), improved FCN with traditional snake (the second column), and our proposed method (the third column). The first row is ED model of subject 45, the second row is ED model of subject 40, and the third row is ES model of subject 30. The color map denotes the mean distance between result mesh and the corresponding reference mesh [3].

Figure 7 shows some segmented LV model from the improved FCN, the improved FCN with traditional snake, and the proposed coarse-to-fine method, respectively. We can see that the proposed method achieved the best segmentation results with the lowest surface distance range. For the improved FCN, due to some obvious leakages in the apex and shrinks in middle part, the segmentation results are relatively worse. The improved FCN with traditional snake can overcome the problem of leakages and shrinks in some extent. Our proposed method, which integrates improved FCN and 3D snake with regularization of constraint term, can further improve the segmentation results on the situations of leakages and shrinks.

#### 3.4.2. Validation

**Figure 8:**
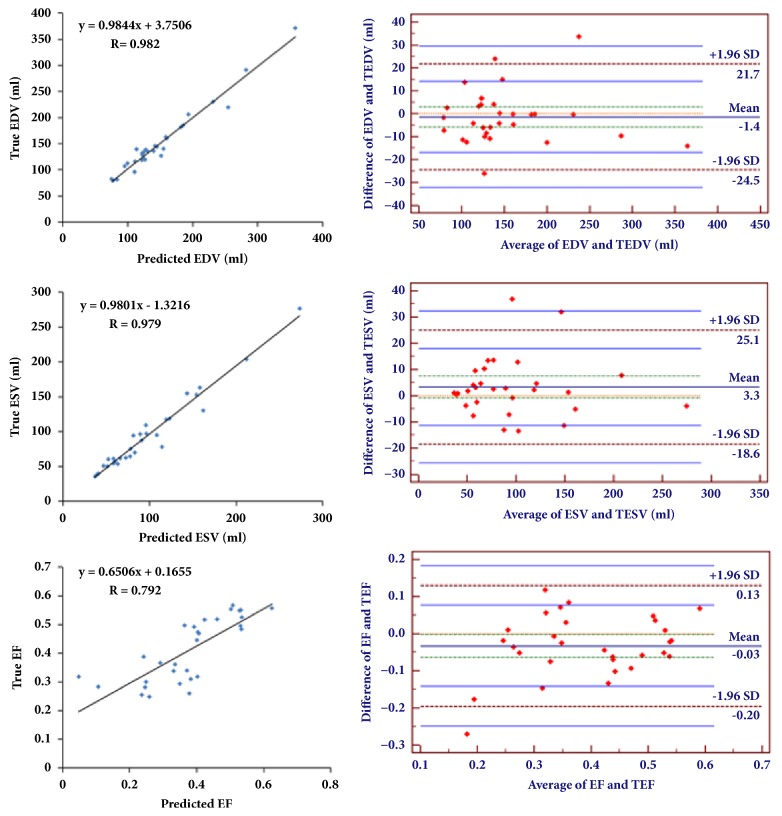
The correlation plots (the left column) and Bland-Altman analysis plots (the right column) for EDV, ESV, and EF on test subjects.

Table 4 and Figure 8 show the correlation between the state-of-the-art segmentation methods (other fully automatic methods and our proposed method) and the ground truth, in terms of EDV, ESV, and EF. The modified correlation values of our proposed method on EDV, ESV, and EF are 0.018, 0.021, and 0.218, respectively, and the corresponding standard deviations are 12 ml, 11.3ml, and 0.07. Table 3 suggests that our proposed method achieves the best performance in EDV and ESV comparing with the state-of-the-art methods in the aspect of EF correlation. The gap of EF correlation is because of the tendency of EDV's bias and ESV's bias are different, though EDV and ESV are more accurate. For example, we assume the ground truth of EDV and ESV are 100 ml and 60 ml. We also assume the first evaluated values of EDV and ESV are 105 ml and 65 ml, and the second evaluated values of EDV and ESV are 103 ml and 57 ml. Based on these values, we can obtain the EF of the ground truth: the first and second evaluated values are 0.4, 0.381, and 0.4466, respectively. Hence, though the accuracy of volume estimation is improved, the EF estimation accuracy is not improved. Furthermore, Figure 8 also displays the results of Bland-Altman analysis of the proposed method for EDV, ESV, and EF and the mean bias values are 1.41, 3.3, and -0.03, respectively. And the corresponding confidence intervals of EDV, ESV, and EF are [21.7, -24.5], [25.1, -18.6], and [0.13, -0.2]. These results indicate the high agreement between our method and ground truth and the big potential of clinical application.

## 4. Discussion

In this paper, we proposed a fully automatic LV segmentation method, which is a coarse-to-fine framework based on the deep FCN and deformable model. In the case of insufficient training datasets, we successfully applied the transfer learning technology combined with the improved FCN for the coarse segmentation of 3DE. The experimental results suggested that the FCN model on the domain of nature image can be successfully transferred to the field of echocardiography images segmentation. This is due to the fact that the low-frequency features, such as edge information and texture information, of VOC images and CETUS images, are the same. Hence, the knowledge of low-frequency feature representations was successfully transferred from VOC images to CETUS images. Otherwise, the added skip streams with residual modules using identity mapping, which fuses different level features from different layers, can make contribution to rapid convergence and high accuracy. This is because the features are different from different layers. Adding the skip streams means that the network has more paths. Hence, adding the skip streams is able to add more different level features to improve the speed of convergence and the accuracy of segmentation. Furthermore, we used the modified loss function, which can solve the problem of data imbalance to improve the segmentation accuracy. As we can see in Figure 7 and Table 3, though the improved FCN improves the coarse segmentation performance and outperform the traditional LV segmentation method, the fine segmentation achieves even better LV segmentation performance. Hence, in Section 2.2, we proposed a 3D LV initialization method based on the coarse segmentation results. As we all know, a good initialization is critical for deformable segmentation model; the results in Table 2 also prove that the proposed 3D LV initialization outperforms the standard initialization method for LV segmentation on 3DE on the case of using the same deformable model.

Additionally, due to the fact that left atrium and left ventricle have the similar intensity value and the presence of papillary muscles, the leakages and shrinks often happen if using the traditional deformable model without the spatial regularization. In this aspect, the proposed 3D snake model with the spatial regularization can improve the final segmentation performance through limiting the free degree of deformation of 3D snake. The results in Section 3 also prove that the proposed method outperforms the state-of-the-art methods for this task on most of the measure indexes.

Though the coarse segmentation results cannot be directly used for clinical application, this attempt shows the potential of end-to-end FCN for LV segmentation task on 3DE. The main limitation of coarse segmentation using FCN is the insufficient training data, which results in that the LV segmentation task on 3DE by end-to-end FCN would not be possible in any time soon. In the future, if more 3DE datasets are accessibly open, the purely end-to-end FCN with superior performance for LV segmentation tasks may be possible and will generate surprising results.

Additionally, 3D snake was used and considered as a postprocessing for the fine segmentation, like conditional random fields (CRF) which is widely used as postprocessing of the natural image segmentation [55–57]. Compared to the purely end-to-end deep learning method, the coarse-to-fine framework has high interpretability [58, 59]. In this paper, the interpretability means that the final segmentation step can be represented by obvious formula, not just the end-to-end deep learning technology. The segmentation performance and model's interpretability are both important for practical clinical application. In the future, we will also study how to formulate the coarse-to-fine frameworks into an end-to-end optimization model to further improve the segmentation performance, at the same time maintaining the interpretability of the coarse-to-fine framework.

## 5. Conclusion

This paper proposed a new fully automatic method for LV segmentation of 3DE based on a coarse-to-fine framework. The proposed FCN, based on transfer learning and residual modules, was used for initial segmentation. From the initial segmentation, an initial model was automatically constructed. The initial model was then used as a constraint item in the energy function of the 3D snake for fine segmentation. The segmentation accuracy and clinical performance (it means the clinical measure results by the proposed method in a true clinical setting) have demonstrated that the proposed method is accurate and has outperformed the state-of-the-art methods on most of the clinical measure indexes. To our best knowledge, we were the first to use a FCN to address the 3DE segmentation problem. This attempt shows the potential of an end-to-end FCN for LV segmentation of 3DE. Besides, the proposed fine segmentation method based on a deformable model not only further improves the segmentation performance, but also provides interpretability for the final segmentation results.

The clinical evaluation suggests that the proposed method has potential for clinical application and may lead to a wide use of FCN in ultrasound image segmentation tasks. In addition, the proposed framework is flexible and can be extended into other applications. In the future, more work about the only pure end-to-end deep learning technology or end-to-end learning framework which combines the deep learning and postprocessing method (which has high interpretability) for segmentation tasks on 3DE will be studied.

## Figures and Tables

**Table 1:** Segmentation accuracy (pixel accuracy (acc), mean accuracy (mean acc), and mean IOU) of different types of FCN in the stage of coarse segmentation in validation set.

The type of networks	Acc	Mean acc	Mean IOU
FCN	0.874	0.853	0.57
U-net	0.854	0.79	0.59
U-net+T	0.91	0.83	0.75
FCN+T	0.9810	0.9678	0.8485
FCN+T+P_(3&4)_	0.984	0.969	0.867
FCN+T+P_(2&3&4)_	0.989	0.973	0.889
FCN+T+P_(1&2&3&4)_	**0.991**	**0.981**	**0.918**

**Table 2:** The segmentation results of different initialization methods with traditional snake on 3DE in test set.

Method	ED						ES					
	d_m_		d_h_		dice		d_m_		d_h_		dice	
	mean	std	mean	std	mean	std	mean	std	mean	std	mean	std
Standard initialization + traditional snake	4.9	1.7	15.9	5.1	0.23	0.18	5.8	1.35	13.5	7.9	0.35	0.15
FCN + traditional snake	4.6	1.58	13.8	4.7	0.29	0.17	5.5	1.67	12.9	7.2	0.38	0.19
FCN+T + traditional snake	3.8	1.34	11.7	4.5	0.26	0.15	4.9	1.27	11.4	5.8	0.34	0.16
FCN+T+P_(3&4)_+ traditional snake	3.35	0.89	9.8	4.2	0.23	0.13	3.48	0.91	10.1	4.9	0.30	0.14
FCN+T+P_(2&3&4)_+ traditional snake	3.15	0.78	9.4	3.7	0.18	0.11	3.1	0.83	9.87	4.2	0.21	0.11
FCN+T+P_(1&2&3&4)_+ traditional snake	**2.9**	**0.7**	**8.9**	**3.1**	**0.13**	**0.008**	**2.8**	**0.75**	**9.3**	**3.9**	**0.16**	**0.05**

**Table 3:** The comparison results of the proposed methods and the five state-of-the-art segmentation methods (Barbosa et al. [28], Milletari et al. [30], van Stralen et al. [31], Smistad et al. [40], and Keraudren et al. [41]) on 3DE in test set.

Method	ED						ES					
	d_m_		d_h_		dice		d_m_		d_h_		dice	
	mean	std	mean	std	mean	std	mean	std	mean	std	mean	std
[28]	2.26	0.73	**8.10**	**2.66**	0.106	0.041	2.43	0.91	**8.13**	3.08	0.144	0.057
[30]	2.14	0.68	8.25	3.87	0.107	0.031	2.91	1.01	8.53	**2.30**	0.162	0.062
[31]	2.44	0.91	8.45	3.50	0.121	0.054	2.79	1.24	8.65	2.85	0.165	0.079
[40]	2.62	0.95	8.26	2.98	0.115	0.038	2.92	0.93	8.99	2.98	0.156	0.050
[41]	2.44	0.95	8.98	3.09	0.130	0.048	2.54	0.75	9.15	3.24	0.158	0.057
Standard initialization + improved snake	4.7	1.76	13.2	4.9	0.22	0.15	4.9	1.7	11.2	5.7	0.25	0.16
improved FCN	3.1	0.8	8.7	3.2	0.17	0.09	2.9	0.57	9.56	3.6	0.21	0.07
improved FCN+ level set	3.2	0.6	9.1	2.9	0.11	0.07	2.5	0.57	9.9	3.7	0.17	0.04
improved FCN+ improved snake	**2.03**	**0.41**	8.80	3.69	**0.098**	**0.0007**	**2.35**	**0.63**	9.09	3.42	**0.125**	**0.0008**

**Table 4:** The comparison with the state-of-the-art methods (Barbosa et al [28], Milletari et al. [30], van Stralen et al. [31], Smistad et al. [40], and Keraudren et al. [41]) on correlation between the segmentation results and the ground truth in test set.

Method	EDV			ESV			EF		
	Corr*∗*	bias	std	Corr*∗*	bias	std	Corr*∗*	bias	std
	val	ml.	ml.	val	ml.	ml.	val	val	val
[28]	0.035	-5	17.7	0.033	-6.8	13.9	**0.111**	**2.9**	5.2
[30]	0.047	5.1	19.0	0.040	-16.8	15.2	0.255	15.2	7.6
[31]	0.034	-15.4	16.0	0.036	-13.2	14.4	0.389	3.7	8.8
[40]	0.049	-10.1	19.4	0.036	-11.3	14.6	0.121	3.7	5.2
[41]	0.079	15.9	24.6	0.048	-6.2	16.6	0.281	12.1	10.6
The proposed method	**0.018**	**1.41**	**12**	**0.021**	**-3.59**	**11.3**	0.208	3.52	**0.07**
